# Explainable diffusion–perfusion radiomics using apparent diffusion coefficient and arterial spin labeling for ATRX status prediction in glioblastoma

**DOI:** 10.3389/fonc.2026.1918991

**Published:** 2026-09-03

**Authors:** Rafail C. Christodoulou, Revati Natu, Zinovia Fakidi, Georgios Vamvouras, Platon S. Papageorgiou, Evros Vassiliou, Elena E. Solomou, Sokratis G. Papageorgiou, Michalis F. Georgiou

**Affiliations:** 1 Division of Neuroimaging and Neurointervention, Department of Radiology, Stanford University, Stanford, CA, United States; 2 Department of Information Technology, K. J. Somaiya School of Engineering, Somaiya Vidyavihar University, Mumbai, India; 3 Faculty of Medicine, Ovidius University of Constanta, Constanta, Romania; 4 Department of Electrical and Computer Engineering, National Technical University of Athens NTUA, Athens, Greece; 5 Department of Medicine, National and Kapodistrian University of Athens, Athens, Greece; 6 Department of Biological Sciences, Kean University, Union, NJ, United States; 7 Internal Medicine-Hematology, University of Patras Medical School, Patras, Greece; 8 1st Department of Neurology, Medical School, National and Kapodistrian University of Athens, Eginition Hospital, Athens, Greece; 9 Department of Radiology, University of Miami, Miami, FL, United States

**Keywords:** apparent diffusion coefficient, ASL MRI, ATRX, biomarker prediction, explainable AI

## Abstract

**Introduction:**

ATRX status serves as a key biomarker for the molecular profiling of glioblastoma, but its evaluation currently depends on invasive tissue sampling. While radiomics has demonstrated promising potential in the molecular analysis of gliomas, the utility of physiological MRI biomarkers for ATRX prediction remains underexplored. Our goal was to develop and assess an explainable combined model utilizing the apparent diffusion coefficient (ADC) and arterial spin labeling (ASL) for the noninvasive prediction of ATRX.

**Methods:**

We retrospectively included 106 patients with histologically confirmed glioblastoma from the UCSD-PTGBM cohort. Radiomics features were extracted from ADC and ASL imaging sequences and used to create a combined machine learning model. The model's performance was assessed using metrics including the area under the receiver operating characteristic curve (AUC), precision-recall analysis, balanced accuracy, calibration metrics, and decision curve analysis. SHapley Additive exPlanations (SHAP) were employed to identify the imaging features most influential to model predictions.

**Results:**

Our integrated ADC and ASL model achieved an AUC of 0.711, with sensitivity 0.737, balanced accuracy 0.713, specificity 0.690, and NPV 0.923 (driven in part by the low prevalence of ATRX loss in the cohort). SHAP analysis revealed that ADC- and ASL-derived features accounted for 48.5% and 51.5% of total mean absolute SHAP importance, respectively, indicating that features from both modalities contributed to predictions within the jointly fitted model, while their independent incremental value remains to be established. Decision curve analysis showed a net benefit at threshold probabilities of roughly 0.01 to 0.46.

**Discussion:**

These findings suggest that combining diffusion and perfusion radiomics can provide noninvasive insight into ATRX status in glioblastoma, though the model's moderate calibration and modest positive predictive value indicate it is best regarded as a hypothesis-generating screening adjunct rather than a diagnostic tool. Radiomics-based machine learning models from physiological MRI may offer valuable insights into ATRX status in glioblastoma; further validation with larger, independent patient cohorts is warranted.

## Graphical Abstract

**Figure:**
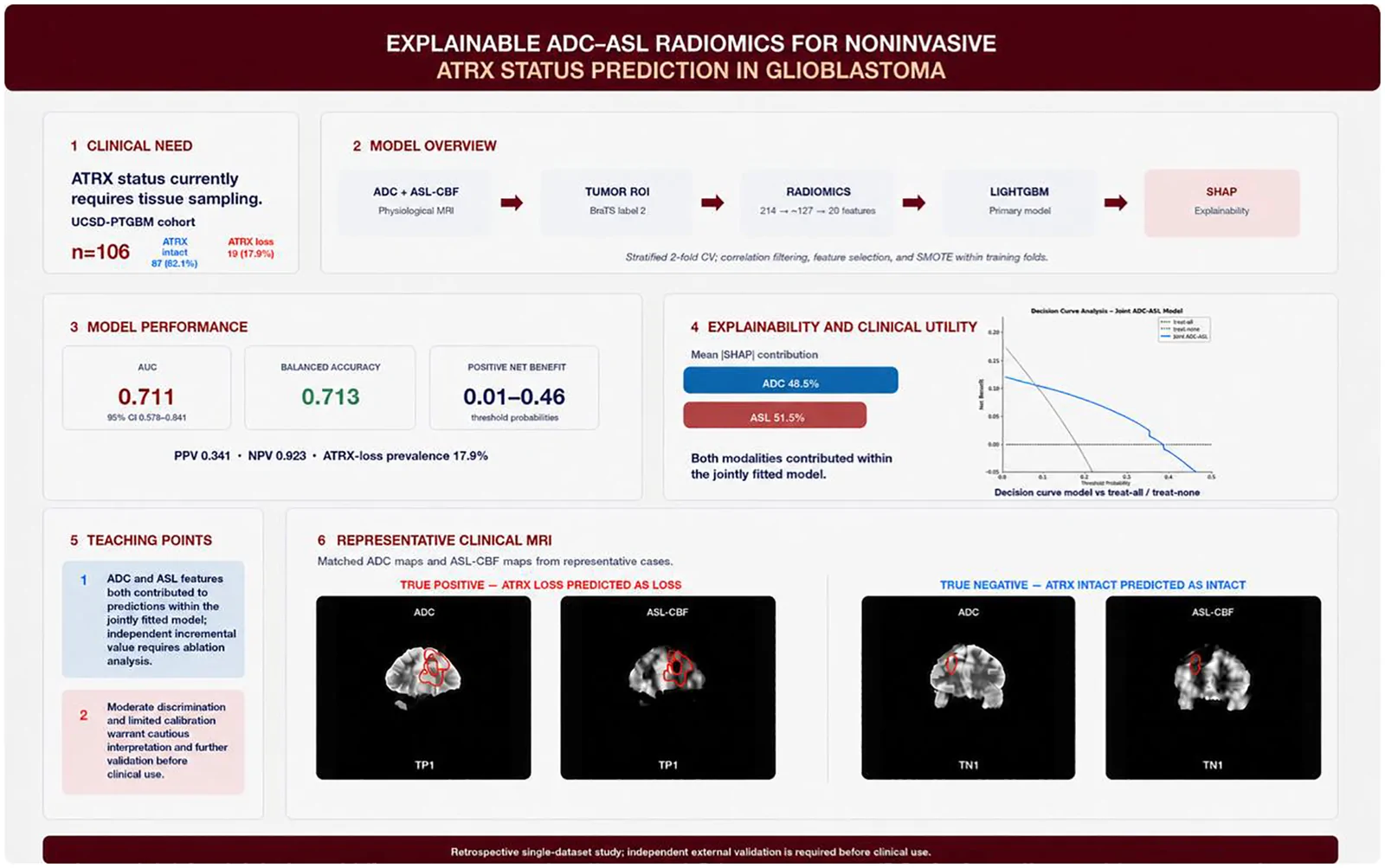

## Introduction

Glioblastoma (GBM) is the most common and aggressive primary malignant brain tumor in adults and remains associated with poor prognosis despite advances in multimodal therapy (1). Current standard treatment includes maximal safe surgical resection combined with temozolomide (TMZ)-based chemoradiation and adjuvant TMZ chemotherapy (2). GBM exhibits substantial molecular, histopathologic, and radiographic heterogeneity, contributing to its variable biological behavior and therapeutic resistance (3). Among the molecular alterations implicated in diffuse glioma biology, ATRX (alpha-thalassemia/mental retardation syndrome X-linked) plays an important role in chromatin remodeling and telomere maintenance (4, 5). ATRX status contributes to glioma classification that goes beyond conventional histology by incorporating molecular characteristics that, at the same time, provide additional biologic and prognostic information about the tumor (6, 7). Despite its clinical relevance, ATRX assessment currently requires invasive tissue sampling followed by molecular or immunohistochemical evaluation (8). Therefore, a non-invasive imaging biomarker capable of predicting ATRX loss preoperatively could provide valuable molecular information while overcoming limitations associated with invasive tissue sampling and intratumoral heterogeneity (9, 10).

Radiomics involves extracting quantitative features from medical images to non-invasively characterize tumor phenotype. These features reveal imaging patterns beyond what is visible to the human eye, including tumor shape, intensity, texture, spatial heterogeneity, and microstructure (10, 11). In glioma imaging, radiomics has been applied for tumor grading and survival prediction. It has also been explored for assessing treatment response, distinguishing recurrence from treatment-related changes, and for molecular characterization (12). Several studies have explored radiomics-based prediction of ATRX status in gliomas (13, 14). A recent systematic review demonstrated promising diagnostic performance of ML-based radiomics in predicting IDH and ATRX alterations in gliomas, while highlighting heterogeneity across studies and MRI protocols (15). Moreover, Meng et al. supported the feasibility of radiomics-based ATRX classification (13), whereas Nacul Mora et al. demonstrated that MRI sequence selection may influence predictive performance, aligning with the fact that each sequence represents distinct biological information that can affect model performance (14).

Although several studies have explored radiomics-based ATRX prediction in gliomas, the combined use of ADC and ASL remains relatively underexplored. ATRX loss in GBM carries distinct biological consequences: it is associated with alternative telomere lengthening, impaired DNA repair, and disrupted cell-cycle regulation, and has been linked to differential sensitivity to temozolomide-based chemoradiation and radiotherapy. Distinguishing ATRX-loss from ATRX-intact GBM preoperatively is therefore biologically and therapeutically relevant — ATRX status may inform molecular risk stratification and guide decisions regarding the intensity of molecular workup and treatment planning. Despite this relevance, ATRX prediction from imaging remains more challenging than IDH or MGMT promoter prediction, with lower reported sensitivities and greater variability across studies, partly because ATRX loss is infrequent in GBM (under 20% of cases) and the imaging phenotype overlaps substantially with ATRX-intact tumors.

Conventional structural MRI sequences — contrast-enhanced T1-weighted, T2-weighted, and FLAIR imaging — capture macroscopic tumor features such as blood-brain barrier disruption, edema, necrosis, and morphology, but offer limited direct insight into tumor microstructure and vascular physiology. ADC maps provide information about tissue diffusion restriction, reflecting cellular density and microstructural organization at a level not visible on structural sequences. ASL-derived cerebral blood flow maps capture tumor perfusion and microvascular density noninvasively and without exogenous contrast, offering mechanistic information about vascular heterogeneity that is orthogonal to structural imaging. The combination of ADC and ASL therefore provides complementary, physiologically grounded information about tumor biology that may capture ATRX-related alterations in diffusion and perfusion heterogeneity beyond what conventional structural MRI can detect. However, the interpretability of current radiomics models remains limited, creating uncertainty in model reasoning and biological interpretation. There is therefore a need for explainable models that both predict ATRX status and identify which imaging features drive those predictions.

We hypothesized that ATRX status in GBM correlates with a distinct diffusion–perfusion radiomic phenotype. Specifically, a joint ADC and ASL radiomics model may capture complementary tumor microstructure and perfusion heterogeneity to predict ATRX loss. To test this hypothesis, we developed a machine-learning radiomics model and used SHAP analysis for explainability, applying ADC and ASL MRI features from histologically confirmed GBM patients.

## Methods

### Study population

This study included 106 patients with histologically confirmed glioblastoma from the UCSD-PTGBM dataset, obtained from The Cancer Imaging Archive (TCIA) (18). ATRX mutation status was dichotomized as Intact (n=87) or Loss (n=19). Age and sex were excluded from all modeling to isolate radiomic signals exclusively.

### MRI acquisition and preprocessing

MRI data were obtained from the UCSD-PTGBM collection on TCIA, acquired at two institutions affiliated with UCSD on 3 Tesla clinical scanners (GE Signa Excite HDx and GE Discovery MR750, both with 8-channel head coil) as part of standard clinical care. Multishell diffusion imaging was acquired using a single-shot pulsed-field gradient spin-echo EPI sequence (TE/TR = 96/17, 000 ms; FOV = 24 cm; matrix = 128×128×48; slice thickness = 2.5 mm) with four b-values (b = 0, 500, 1500, and 4000 s/mm²). ADC maps were derived from this acquisition. ASL was acquired using the standard GE pCASL protocol with in-line CBF map generation. Images were provided at 1×1×1 mm isotropic resolution; no additional resampling was performed during feature extraction. Detailed per-subject acquisition parameters are available in the TCIA collection metadata. ADC maps were derived from diffusion-weighted imaging using a b-value of 4000 s/mm² and were provided as preprocessed maps within the UCSD-PTGBM dataset. ASL cerebral blood flow maps were generated using pseudo-continuous arterial spin labeling (pCASL) sequences and are included as quantitative CBF maps. The pCASL was acquired using the GE scanner protocol. No additional inter-subject normalization of ADC or ASL maps was applied beyond the per-image intensity normalization performed by PyRadiomics during feature extraction. Images were provided at 1×1×1 mm isotropic resolution as part of the UCSD-PTGBM dataset preprocessing pipeline; no additional resampling was applied during feature extraction. Feature extraction was performed using PyRadiomics (version 3.1.1). A fixed bin width of 25 was applied for intensity discretization, consistent with established PyRadiomics workflows.

### Tumor segmentation and region of interest

Tumor segmentation masks were obtained from the UCSD-PTGBM collection on TCIA. Within this cohort, a subset of patients underwent manual expert segmentation by qualified neuroradiologists, while the remainder were pre-annotated using the BraTS 2024 deep learning segmentation network and subsequently reviewed and refined by expert readers, following the BraTS 2024 protocol and label conventions throughout. Radiomic features were extracted from label 2 of the segmentation mask, corresponding to the contrast-enhancing tumor subregion as defined by the BraTS 2024 convention. This subregion was selected as it represents the most biologically active tumor compartment and has demonstrated relevance in prior GBM radiomic studies. Segmentation masks from both sources were generated in accordance with the BraTS 2024 protocol, ensuring consistent tumor subregion definitions and label conventions across the entire cohort.

### MRI sequences and feature extraction

Radiomic features were extracted from two functional MRI sequences — apparent diffusion coefficient (ADC) maps, reflecting cellular diffusion restriction and microstructural organization, and arterial spin labeling (ASL) maps, reflecting cerebral perfusion and microvascular density. Feature extraction was performed using PyRadiomics with standardized preprocessing. A combined feature matrix of 214 features was obtained prior to any selection step.

### Preprocessing

**Figure 1:**
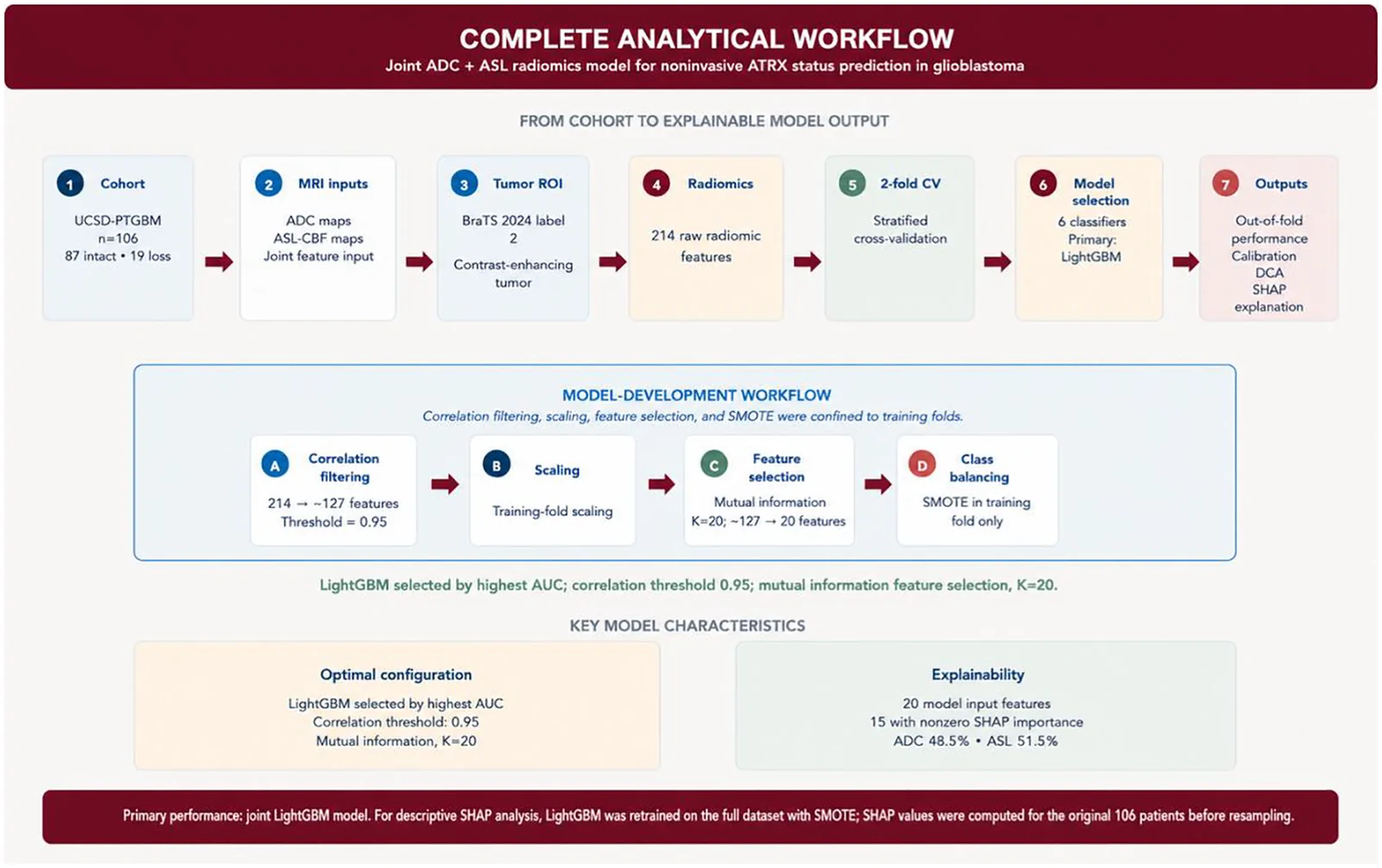
Complete analytical workflow for the joint ADC+ASL radiomics model. The diagram summarizes cohort selection, MRI inputs, contrast-enhancing tumor ROI definition, radiomic feature extraction and reduction, stratified 2-fold cross-validation, training-fold processing, model selection, performance evaluation, and descriptive SHAP analysis.

Correlation-based filtering was applied to remove redundant features, with six thresholds evaluated (0.85, 0.90, 0.95, 0.97, 0.98, 0.99). Correlation coefficients were computed exclusively on training-fold data within each cross-validation iteration, and features exceeding the threshold were removed prior to any feature scaling or selection. This ensured that correlation filtering, like all subsequent preprocessing steps, was performed without access to test-fold information. All feature scaling was performed using StandardScaler fitted exclusively on training folds to prevent data leakage. Missing values and infinite values were replaced with zero prior to processing (**Figure 1**).

### Feature selection

**Figure 2:**
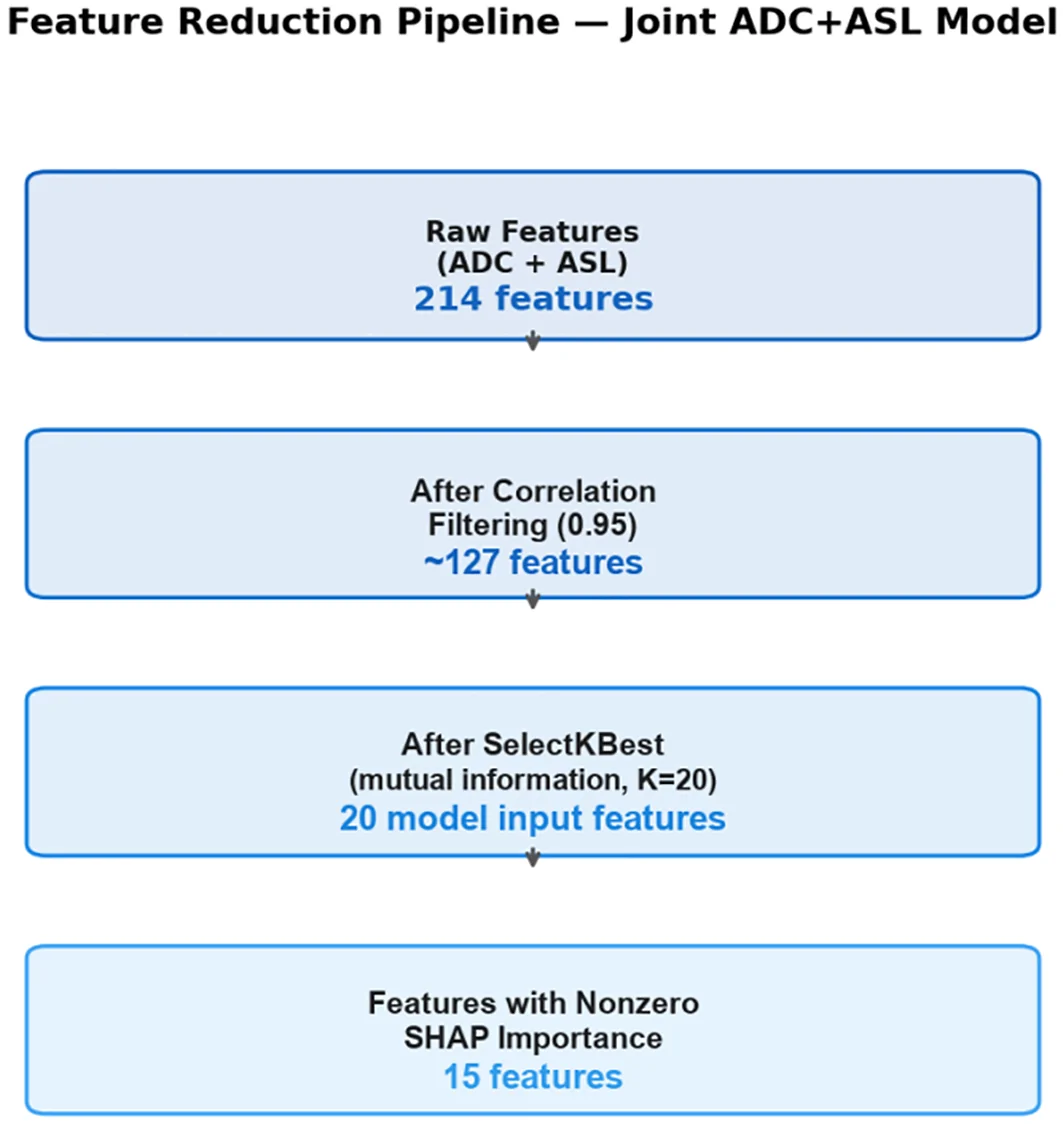
Feature reduction pipeline for the joint ADC+ASL model, illustrating the sequential reduction from 214 raw features to approximately 127 (after correlation filtering, threshold 0.95), then to 20 model input features (after mutual information-based SelectKBest, K = 20). Of the 20 model inputs, 15 received nonzero SHAP importance in the fitted LightGBM model.

**Figure 3:**
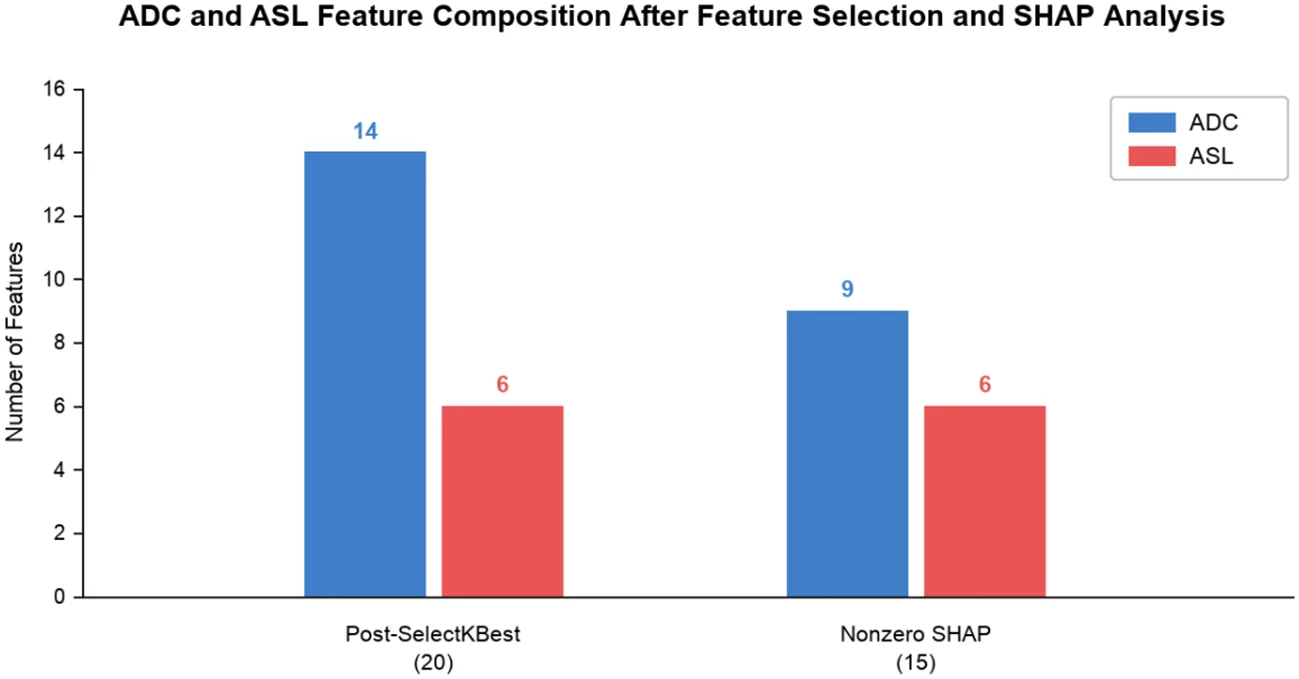
ADC and ASL feature composition among the 20 model input features selected by mutual information-based SelectKBest (K = 20) and the 15 features with nonzero SHAP importance in the fitted LightGBM model. Of the 20 model inputs, 14 were ADC-derived and 6 were ASL-derived; among the 15 features with nonzero SHAP importance, 9 were ADC-derived and 6 were ASL-derived.

SelectKBest was applied using two scoring functions, evaluated in parallel: ANOVA F-statistic and mutual information. Mutual information was included alongside ANOVA to capture nonlinear relationships between radiomic features and ATRX mutation status that linear scoring alone may miss. K values of 5, 8, 10, 12, 15, 20, 32, 50, 64, and 80 were evaluated. All feature selection was performed strictly within each cross-validation fold to prevent leakage. The sequential feature reduction process is illustrated in **Figures 2** and **3**. The combined feature matrix was reduced from 214 raw features to approximately 127 after correlation filtering (threshold 0.95), and then to 20 model input features after mutual information-based selection (K = 20). Of these 20 model inputs, 15 received nonzero SHAP importance in the fitted LightGBM model; the remaining 5 ADC features were assigned zero weight by LightGBM.

### Class imbalance

The dataset exhibited significant class imbalance, with ATRX Intact cases comprising 82.1% of the cohort. Synthetic Minority Oversampling Technique (SMOTE) was applied within each training fold only to address this imbalance. The number of nearest neighbours was set to min(3, n_minority − 1) to accommodate the small Loss class. SMOTE was not applied to test folds at any stage.

### Cross-validation

Stratified 2-fold cross-validation was used throughout, preserving ATRX-status proportions across folds. This configuration was selected to maximize training fold size given the limited cohort (n=106) and pronounced class imbalance (19 ATRX-loss cases, ~18%); a 5-fold strategy would yield approximately 3–4 minority-class samples per test fold, precluding stable minority-class performance estimation. Given the limited cohort size, stratified 2-fold cross-validation was selected to preserve adequate minority-class representation in each test partition while maximizing the availability of training data. Fold composition was examined for sex balance: Fold 1 contained 37 male and 16 female patients, and Fold 2 contained 37 male and 16 female patients, confirming proportional sex distribution across folds. ATRX-loss cases were distributed as 10 and 9 across Fold 1 and Fold 2 respectively, consistent with stratified sampling. Out-of-fold probabilities were collected for all 106 patients to generate performance estimates across the full cohort. Correlation-based filtering, feature scaling (StandardScaler), feature selection (SelectKBest), and SMOTE were each applied exclusively within training folds during cross-validation. Because model configuration selection — including correlation threshold, feature-selection scorer, and feature count K — was performed across the same two folds subsequently used to report the final AUC, the reported metrics reflect the best-identified configuration and may carry some optimistic bias relative to a fully nested framework.

### Classifiers

Six classifiers were evaluated: Gradient Boosting (n_estimators=300, learning_rate=0.05, max_depth=3, subsample=0.8), LightGBM (n_estimators=300, learning_rate=0.05, num_leaves=15, min_child_samples=5, class_weight=balanced), Logistic Regression (C = 0.1, solver=saga, class_weight=balanced), Random Forest (n_estimators=500, max_features=sqrt, min_samples_leaf=2, class_weight=balanced), Support Vector Machine (RBF kernel, C = 1.0, class_weight=balanced), and K-Nearest Neighbours (k=5). All classifiers were evaluated across the full grid of correlation thresholds, feature selection scorers, and k values. The optimal joint model was identified as LightGBM trained at a correlation threshold of 0.95, using mutual information-based feature selection with K = 20, based on achieving the highest AUC across the cross-validation grid search. LightGBM was therefore designated as the primary model for all reported performance metrics and SHAP explainability analysis. The rank-based ensemble result is reported separately as an exploratory estimate and is not used as the basis for primary performance claims. Classifier hyperparameters were fixed *a priori* based on established defaults and were not tuned within the cross-validation framework; only the correlation threshold, feature-selection scorer, and feature count K were varied across the grid search.

### Joint modeling strategy

ADC and ASL features were combined into a single joint feature matrix prior to correlation filtering and feature selection. This allowed the selection step to identify the most informative features across both sequences simultaneously, capturing cross-sequence complementarity between diffusion and perfusion radiomic signatures rather than treating each sequence independently. The optimal configuration was identified through a systematic grid search across all combinations of correlation threshold, feature selection scorer, k value, and classifier.

### Ensemble strategy

A rank-based ensemble was evaluated as an exploratory approach. Out-of-fold predicted probabilities from each classifier trained on the joint feature matrix were converted to percentile ranks and averaged. Rank averaging was employed to reduce sensitivity to differences in probability calibration between classifiers. The ensemble result is reported as an exploratory upper-bound estimate and not as a primary performance claim.

### Performance evaluation

Model performance was evaluated using the area under the receiver operating characteristic curve (AUC) as the primary metric. The Youden index was used to determine the optimal classification threshold for each model. Bootstrap confidence intervals were computed using 1000 iterations. Additional metrics included sensitivity, specificity, positive predictive value (PPV), negative predictive value (NPV), F1 score, balanced accuracy, precision-recall AUC, and Brier score. Pairwise AUC comparisons were performed using the DeLong test. Decision curve analysis (DCA) was performed to evaluate net clinical benefit across a range of threshold probabilities, benchmarked against treat-all and treat-none strategies.

### Explainability analysis

SHAP (SHapley Additive exPlanations) values were computed for the optimal joint model using a TreeExplainer applied to the trained LightGBM classifier. The model was retrained on the full dataset with SMOTE applied for this analysis. SHAP values were computed for the original 106 patients prior to resampling to ensure interpretability reflects the true patient distribution. Mean absolute SHAP values were used to rank feature importance and quantify the relative contribution of ADC and ASL sequences to model predictions. This approach provides a descriptive interpretation of the final fitted model — the SHAP values characterize feature importance as learned by the model on the full dataset and do not constitute out-of-sample explanations. Accordingly, SHAP results should be interpreted as describing the model’s learned decision patterns rather than as independently validated feature importance estimates.

## Results

### Study cohort

A total of 106 patients were included, of whom 87 (82.1%) had ATRX Intact status and 19 (17.9%) had ATRX Loss, reflecting the known prevalence of ATRX alterations in glioblastoma (**Table 1**).

**Table 1:** Patient demographics and clinical characteristics of the study cohort (n=106).

Characteristic	Joint ATRX intact (n=87)	ATRX loss (n=19)
Age, years — mean (SD)	57.5 (10.2)	49.2 (22.4)
Age, years — median	58.0	54.0
Sex — Male, n (%)	61 (70.1%)	13 (68.4%)
Sex — Female, n (%)	26 (29.9%)	6 (31.6%)

The Youden index was used to determine the optimal classification threshold.

### Model performance

**Figure 4:**
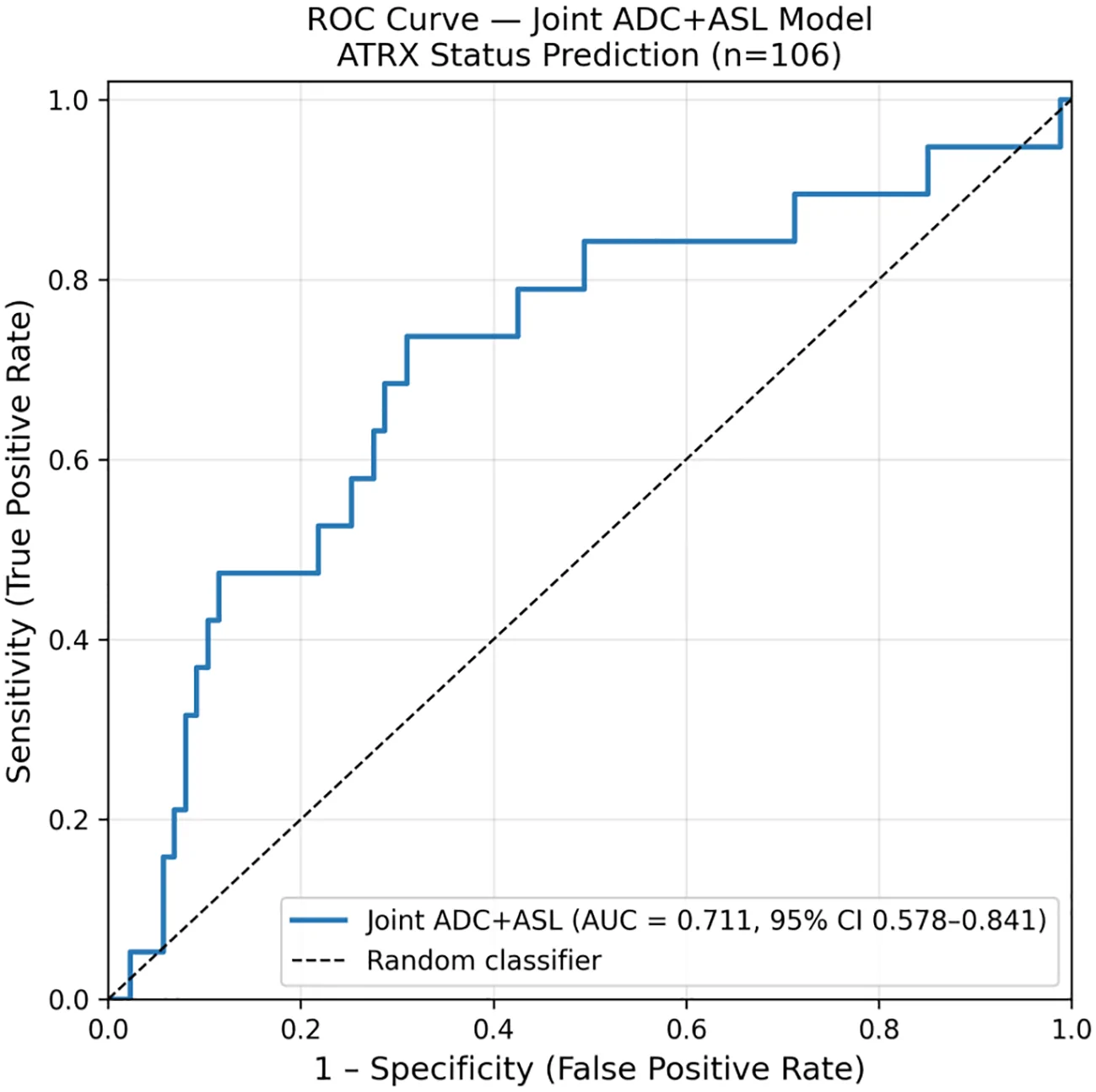
Receiver operating characteristic (ROC) curve for the joint ADC+ASL model predicting ATRX mutation status in glioblastoma (n=106).

The joint ADC+ASL model achieved an AUC of 0.711 (95% CI 0.578–0.841), with a sensitivity of 0.737, specificity of 0.690, PPV of 0.341, NPV of 0.923, F1 score of 0.467, and balanced accuracy of 0.713. The optimal classification threshold determined by the Youden index was 0.074. The Brier score was 0.153. Full performance metrics with bootstrap confidence intervals are presented in **Table 2**, and the ROC curve for the joint model is presented in **Figure 4**.

**Table 2:** Performance metrics for the joint ADC+ASL model with 95% bootstrap confidence intervals (n=1000 iterations).

Metric	Joint ADC+ASL model
AUC	0.711 (0.578–0.841)
Sensitivity	0.737 (0.450–1.000)
Specificity	0.690 (0.488–0.932)
PPV	0.341 (0.210–0.650)
NPV	0.923 (0.860–1.000)
F1 Score	0.467 (0.324–0.651)
Balanced Accuracy	0.713 (0.636–0.830)
PR-AUC	0.318 (0.184–0.530)
Brier Score	0.153 (0.099–0.209)
Optimal Threshold	0.074

The Youden index was used to determine the optimal classification threshold.

The NPV of 0.923 indicates that when the model predicts ATRX Intact status, it is correct in approximately 92% of cases. However, this value is strongly influenced by the low prevalence of ATRX loss in the cohort (17.9%) and should not by itself be taken as evidence of clinical rule-out utility without validation in settings with different prior probabilities. Similarly, the PPV of 0.341 should be interpreted with caution: at the operating threshold, approximately 7 in 10 patients predicted to have ATRX loss would in fact be ATRX-intact, representing a substantial false-positive burden. In settings where a positive prediction triggers invasive molecular workup or alters clinical management, this rate of false positives may limit the model’s standalone utility and reinforces its role as a screening adjunct rather than a confirmatory tool.

### Model calibration

**Figure 5:**
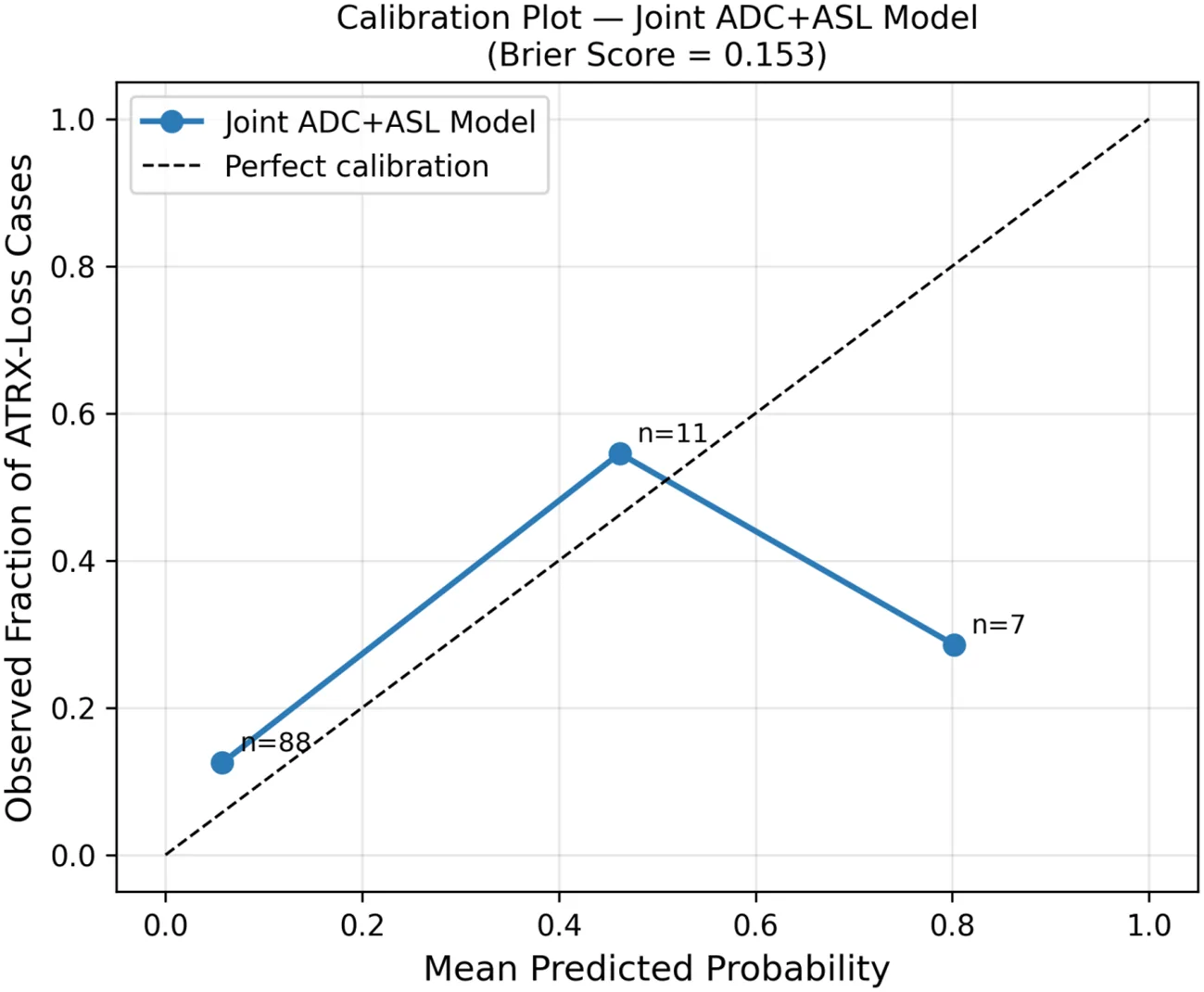
Calibration plot for the joint ADC+ASL model, showing the relationship between mean predicted probability and observed fraction of ATRX-loss cases across three uniform probability bins (Bin 1: n=88; Bin 2: n=11; Bin 3: n=7; total n=106). The Brier score was 0.153. The dashed line represents perfect calibration.

The Brier score for the joint ADC+ASL model was 0.153, reflecting moderate probabilistic accuracy in the context of the cohort’s class imbalance (18% ATRX-loss prevalence). A calibration plot is presented in **Figure 5**. Three uniform probability bins were used, accounting for all 106 patients. In the low-probability bin (predicted probability 0.00–0.33; mean predicted probability 0.058, n=88), the observed fraction of ATRX-loss cases was 0.125; in the intermediate bin (0.33–0.67; mean predicted probability 0.462, n=11), the observed fraction was 0.545; and in the high-probability bin (0.67–1.00; mean predicted probability 0.802, n=7), the observed fraction was 0.286. This pattern indicates that the model’s predicted probabilities are not well-calibrated and should not be used directly as risk estimates without recalibration. These calibration characteristics should be considered alongside the discrimination metrics when evaluating the model’s suitability for clinical probability-based decision-making.

### Clinical utility

**Figure 6:**
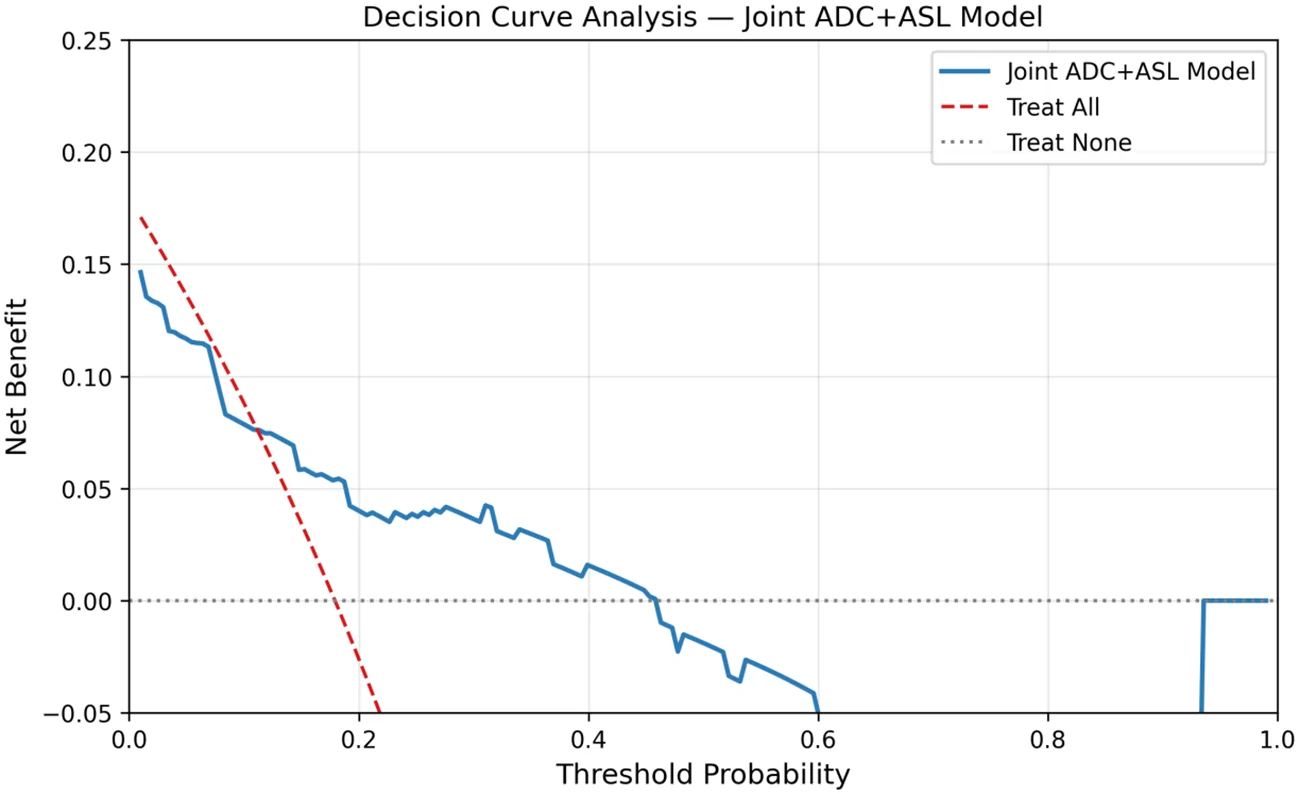
Decision curve analysis for the joint ADC+ASL model demonstrating net clinical benefit across a range of threshold probabilities, benchmarked against treat-all and treat-none strategies.

Decision curve analysis demonstrated that the joint ADC+ASL model maintained positive net clinical benefit across a threshold probability range of approximately 0.01 to 0.46, outperforming the treat-none strategy across this range, as shown in **Figure 6**. This suggests the model may provide some decision support across a clinically plausible range of pre-test probability settings for ATRX Loss in glioblastoma; however, given the model’s acknowledged poor calibration, these findings should be interpreted with caution and net benefit estimates may not translate reliably to settings with different prevalence or threshold assumptions. Outside the 0.01–0.46 range the model provided little to no net benefit over the treat-all and treat-none reference strategies respectively, and this range limitation should be considered a constraint on the current model’s practical decision-support scope.

### SHAP explainability

**Figure 7:**
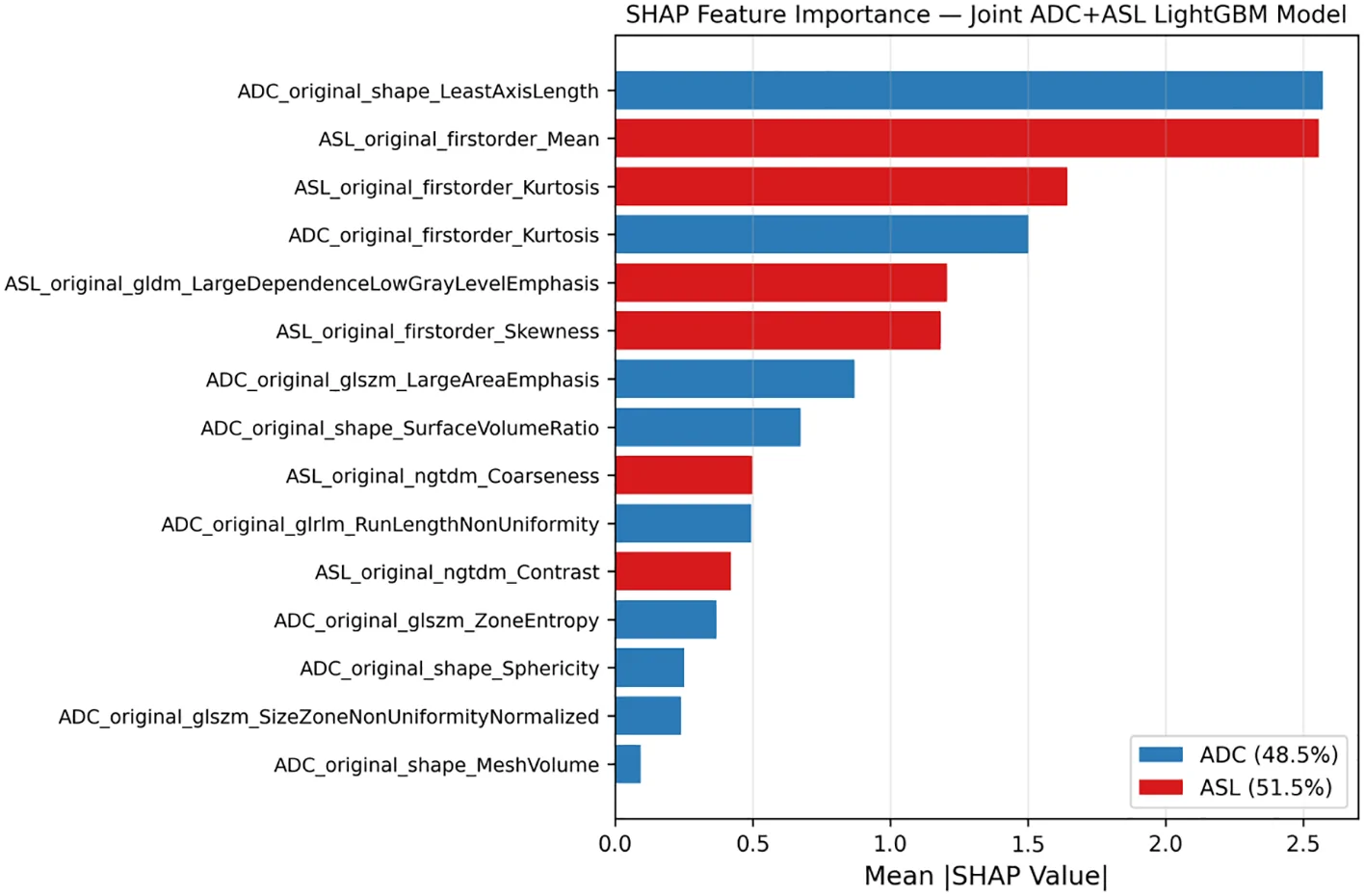
SHAP feature importance summary for the joint ADC+ASL LightGBM model. Features are ranked by mean absolute SHAP value. Blue bars represent ADC-derived features and red bars represent ASL-derived features.

SHAP analysis of the joint ADC+ASL model demonstrated near-equal contributions from diffusion and perfusion features, with ADC accounting for 48.5% and ASL accounting for 51.5% of total mean absolute SHAP importance within the jointly fitted model. These proportions reflect relative feature importance as learned by the model and are consistent with both sequences contributing to predictions; however, they do not establish statistical independence or incremental improvement of one modality over the other, as no ablation or single-modality comparison was performed. The ranked SHAP feature importance values are presented in **Table 3**, and the SHAP summary plot is presented in **Figure 7**.

**Table 3:** Top radiomic features ranked by mean absolute SHAP value for the joint ADC+ASL LightGBM model.

Rank	Sequence	Feature	Category	Mean │SHAP│
1	ADC	shape_LeastAxisLength	Shape	2.571
2	ASL	firstorder_Mean	First Order	2.556
3	ASL	firstorder_Kurtosis	First Order	1.643
4	ADC	firstorder_Kurtosis	First Order	1.502
5	ASL	gldm_LargeDependenceLowGrayLevelEmphasis	Texture (GLDM)	1.205
6	ASL	firstorder_Skewness	First Order	1.182
7	ADC	glszm_LargeAreaEmphasis	Texture (GLSZM)	0.87
8	ADC	shape_SurfaceVolumeRatio	Shape	0.674
9	ASL	ngtdm_Coarseness	Texture (NGTDM)	0.497
10	ADC	glrlm_RunLengthNonUniformity	Texture (GLRLM)	0.495
11	ASL	ngtdm_Contrast	Texture (NGTDM)	0.42
12	ADC	glszm_ZoneEntropy	Texture (GLSZM)	0.367
13	ADC	shape_Sphericity	Shape	0.251
14	ADC	glszm_SizeZoneNonUniformityNormalized	Texture (GLSZM)	0.239
15	ADC	shape_MeshVolume	Shape	0.092

ADC total contribution: 48.5%. ASL total contribution: 51.5%. Features with zero SHAP importance (n=5) are excluded from this table.

The single most influential feature was ADC-derived LeastAxisLength (mean |SHAP|=2.571), followed by ASL Mean (2.556), ASL Kurtosis (1.643), ADC Kurtosis (1.502), and ASL LargeDependenceLowGrayLevelEmphasis (1.205). Five ADC features — HighGrayLevelRunEmphasis, GrayLevelNonUniformityNormalized, InverseVariance, GrayLevelVariance, and Coarseness — were selected during feature selection but assigned zero SHAP importance by LightGBM, indicating that the model assigned zero weight to these features in the fitted model.

The single most influential feature was ADC-derived LeastAxisLength (mean |SHAP|=2.571), followed by ASL Mean (2.556), ASL Kurtosis (1.643), ADC Kurtosis (1.502), and ASL LargeDependenceLowGrayLevelEmphasis (1.205). Five ADC features were assigned zero SHAP importance by LightGBM in the fitted model, with the remaining 15 features accounting for all nonzero SHAP contributions.

### Representative case examples

**Figure 8:**
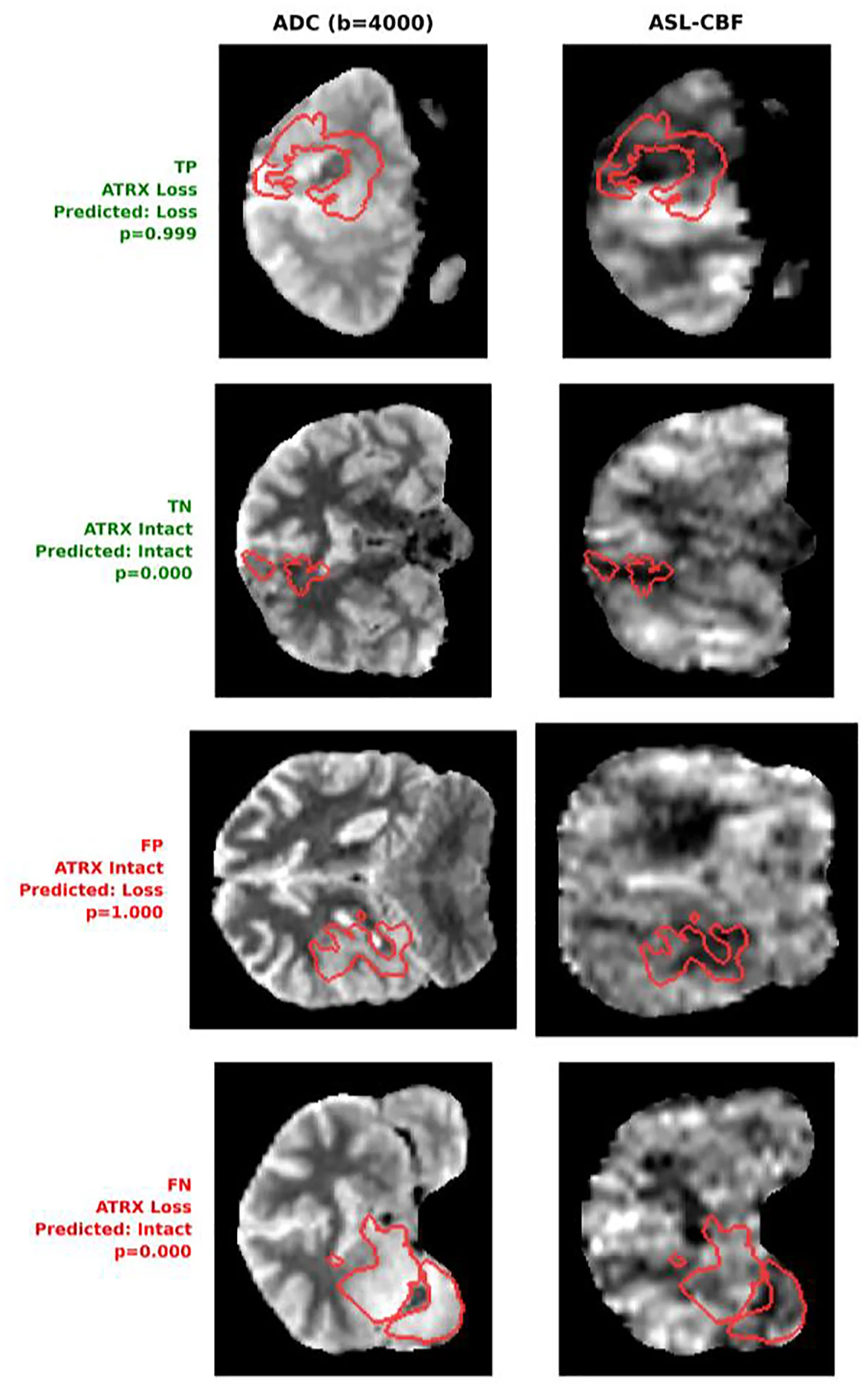
Representative ADC (b=4000) and ASL-CBF images for a true-positive (TP), true-negative (TN), false-positive (FP), and false-negative (FN) case. The red contour delineates the contrast-enhancing tumor subregion (BraTS label 2) used for radiomic feature extraction. Predicted probabilities and true ATRX labels are indicated for each case.

Representative ADC and ASL-CBF images for one case from each prediction category are presented in **Figure 8**. The true-positive case (UCSD-PTGBM-0009_01; ATRX loss, predicted loss; p=0.999) and false-positive case (UCSD-PTGBM-0077_01; ATRX intact, predicted loss; p=1.000) both received high predicted probabilities, suggesting that the model identified similar diffusion and perfusion texture patterns in both cases despite differing molecular status. The true-negative case (UCSD-PTGBM-0108_01; ATRX intact, predicted intact; p=0.000) and false-negative case (UCSD-PTGBM-0097_01; ATRX loss, predicted intact; p=0.000) both received near-zero predicted probabilities. The confident misclassification of the false-negative case highlights that ATRX-loss tumors do not always express a detectable diffusion-perfusion phenotype at the radiomic level, consistent with the known biological heterogeneity of ATRX alterations in GBM. These examples illustrate that prediction errors are biologically plausible and reinforce the hypothesis-generating nature of the current model.

## Discussion

### Principal findings

In this study, we developed an explainable joint radiomics model that combines ADC and ASL to noninvasively predict ATRX status in glioblastoma. The model demonstrated moderate discriminative performance, with an AUC of 0.711, sensitivity of 0.737, specificity of 0.690, NPV of 0.923, balanced accuracy of 0.713, and PR-AUC of 0.318. The NPV of 0.923 should be interpreted with caution: it is substantially driven by the low prevalence of ATRX loss in this cohort (17.9%) and does not by itself constitute evidence of clinical rule-out utility. This finding requires validation in independent cohorts before any clinical inference can be drawn. The results suggest that features from both diffusion and perfusion imaging contributed to predictions within the jointly fitted model, although their independent incremental value remains to be established. The PPV of 0.341 and PR-AUC of 0.318 indicate that positive predictions carry substantial uncertainty — approximately 7 in 10 patients predicted to have ATRX loss would in fact have intact status at the operating threshold. If used to guide clinical decision-making, false-positive predictions would direct ATRX-intact patients toward unnecessary additional molecular workup, while false-negative predictions — classifying ATRX loss as intact — could result in missed molecular information relevant to treatment planning and risk stratification. Given these characteristics, the current model is best understood as a hypothesis-generating screening adjunct rather than a diagnostic tool, and any potential clinical application should be limited to settings where the threshold probability falls within the demonstrated net-benefit range.

### Explainability and biological interpretation

SHAP explainability analysis offered biological insight into the radiomics model predictions by identifying the contributions of diffusion- and perfusion-related features (19). Both ADC and ASL features contributed to model predictions, with SHAP contributions of 48.5% and 51.5%, respectively, within the jointly fitted model. This pattern is descriptively consistent with both sequences contributing to learned predictions; formal ablation comparisons would be required to establish whether each modality provides statistically independent or incremental predictive value. Perfusion features such as GLDM Large Dependence Low Gray Level Emphasis, ASL skewness, and kurtosis suggest that variation in blood-flow patterns and vascularity is associated with ATRX status (16, 20). Diffusion-derived shape features reflecting the spatial characteristics and extent of diffusion within the tumor, rather than purely tumor size, also appeared informative for ATRX prediction, as they may reflect alterations in tumor morphology and growth (3). In particular, ADC kurtosis may reflect differences in cellularity, necrosis, edema, and microstructural complexity, whereas diffusion texture features may capture spatial heterogeneity within the tumor (21, 22). These observations are consistent with previous reports suggesting that advanced MRI can capture biological differences related to the molecular characterization of gliomas (9, 16, 17).

### Comparison with previous studies

Although several studies have explored the use of radiomics for ATRX prediction in gliomas, two of them are particularly relevant for comparison with our findings. Meng et al. used multiparametric MRI that included T1, T2, FLAIR, and ADC in gliomas II-IV and reported a validation AUC of 0.84, while Nacul Mora et al. demonstrated that predictive performance varied substantially depending on the MRI sequences included, with T2-weighted imaging achieving the highest AUC of 0.831 (13, 14). In comparison, our combined ADC and ASL model achieved an AUC of 0.711. This more moderate performance may be attributed to several factors. First, whereas most previous studies included mixed-grade gliomas, our study focused exclusively on glioblastoma, a biologically distinct and more challenging tumor type for ATRX prediction (1, 6). Furthermore, age and sex were intentionally excluded from the analysis, allowing the model to rely exclusively on radiomics features. Finally, we deliberately restricted the model to ADC- and ASL-derived biomarkers in order to investigate whether diffusion and perfusion information alone could predict ATRX status.

### Strengths of the study

Despite the more moderate predictive performance, the proposed model has several important strengths. By focusing exclusively on glioblastoma, biological heterogeneity was reduced, and a more disease-specific evaluation of ATRX status was achieved (1, 6). Second, the model leveraged advanced physiological MRI biomarkers derived from ADC and ASL, which offer direct mechanistic insights into tumor cellularity and microvascular perfusion. Conventional structural sequences, such as T1-weighted post-contrast and FLAIR imaging, primarily capture macroscopic phenomena like blood-brain barrier disruption and infiltrative vasogenic edema. In contrast, ADC and ASL mapping provide a more nuanced characterization of the tumor’s underlying microstructural complexity and hemodynamic physiology, offering an independent physiological profile rather than relying solely on conventional macrostructural changes (16, 20). Third, decision curve analysis demonstrated a positive net benefit across a threshold probability range of approximately 0.01–0.46, outperforming the treat-none strategy across this range (23). Last but not least, several methodological precautions were implemented to minimize data leakage, including performing feature selection within cross-validation folds. In addition, scaling parameters were fitted exclusively to the training data, and SMOTE was applied only to the training folds (24).

### Limitations and future directions

Several limitations should be acknowledged. First, this was a retrospective single-dataset study, which may have introduced selection bias and may limit the generalizability of the findings. Second, the limited number of ATRX-loss cases necessitated 2-fold cross-validation, which may have yielded less stable estimates than would be expected in larger cohorts. Third, no external validation cohort was available, preventing assessment of model robustness across institutions, scanners, and acquisition protocols. Finally, SHAP analysis suggested that some imaging features had limited incremental information; however, these exploratory findings should be interpreted with caution and not overemphasized. Future studies should validate these findings in larger, independent cohorts and further compare physiological MRI-based models with conventional MRI approaches. Model configuration selection was not performed within a fully nested cross-validation framework; the correlation threshold, feature-selection scorer, and feature count K were chosen based on performance from the same folds used to estimate the final AUC. This may introduce optimistic bias, and the results should be interpreted as exploratory performance estimates rather than strictly unbiased generalization bounds.

## Conclusion

Our study shows that combining ADC and ASL radiomics can provide insights into ATRX status in glioblastoma. Features from both diffusion and perfusion contributed to model predictions, suggesting that ADC and ASL may capture complementary aspects of tumor biology relevant to ATRX status; whether each modality provides independent incremental value requires dedicated ablation analysis. SHAP analysis enhanced understanding by highlighting the imaging features most influential to the model’s predictions. These findings are preliminary and need validation with larger, independent groups. They should be viewed in light of the retrospective nature, the small ATRX-loss sample, the use of 2-fold cross-validation, and the absence of external validation. Still, the results suggest that physiological MRI radiomics has potential for non-invasive molecular profiling of glioblastoma. Future studies should compare physiological MRI radiomics with conventional MRI and explore multimodal approaches that combine imaging with clinical data.

## Data Availability

Publicly available datasets were analyzed in this study. This data can be found here: https://www.cancerimagingarchive.net/collection/ucsd-ptgbm/ DOI: 10.7937/fwv2-dt74.

## References

[1] Schaff LR Mellinghoff IK . Glioblastoma and other primary brain Malignancies in adults: A review. JAMA. (2023) 329:574–87. doi: 10.1001/jama.2023.0023 36809318 PMC11445779

[2] Weller M Van Den Bent M Preusser M Le Rhun E Tonn JC Minniti G . EANO guidelines on the diagnosis and treatment of diffuse gliomas of adulthood. Nat Rev Clin Oncol. (2021) 18:170–86. doi: 10.1038/s41571-020-00447-z 33293629 PMC7904519

[3] Becker AP Sells BE Haque SJ Chakravarti A . Tumor heterogeneity in glioblastomas: From light microscopy to molecular pathology. Cancers. (2021) 13:761. doi: 10.3390/cancers13040761 33673104 PMC7918815

[4] Aguilera P López-Contreras AJ . ATRX, a guardian of chromatin. Trends Genet. (2023) 39:505–19. doi: 10.1016/j.tig.2023.02.009 36894374

[5] Pang Y Chen X Ji T Cheng M Wang R Zhang C . The chromatin remodeler ATRX: Role and mechanism in biology and cancer. Cancers. (2023) 15:2228. doi: 10.3390/cancers15082228 37190157 PMC10136513

[6] Louis DN Perry A Wesseling P Brat DJ Cree IA Figarella-Branger D . The 2021 WHO classification of tumors of the central nervous system: A summary. Neuro-Oncol. (2021) 23:1231–51. doi: 10.1093/neuonc/noab106 34185076 PMC8328013

[7] Nandakumar P Mansouri A Das S . The role of ATRX in glioma biology. Front Oncol. (2017) 7. doi: 10.3389/fonc.2017.00236 29034211 PMC5626857

[8] Ikemura M Shibahara J Mukasa A Takayanagi S Aihara K Saito N . Utility of ATRX immunohistochemistry in diagnosis of adult diffuse gliomas. Histopathology. (2016) 69:260–7. doi: 10.1111/his.12927 26741321

[9] Kickingereder P Bonekamp D Nowosielski M Kratz A Sill M Burth S . Radiogenomics of glioblastoma: Machine learning–based classification of molecular characteristics by using multiparametric and multiregional MR imaging features. Radiology. (2016) 281:907–18. doi: 10.1148/radiol.2016161382 27636026

[10] Gillies RJ Kinahan PE Hricak H . Radiomics: Images are more than pictures, they are data. Radiology. (2016) 278:563–77. doi: 10.1148/radiol.2015151169 26579733 PMC4734157

[11] Sala E Mema E Himoto Y Veeraraghavan H Brenton JD Snyder A . Unravelling tumour heterogeneity using next-generation imaging: Radiomics, radiogenomics, and habitat imaging. Clin Radiol. (2017) 72:3–10. doi: 10.1016/j.crad.2016.09.013 27742105 PMC5503113

[12] Zhou M Scott J Chaudhury B Hall L Goldgof D Yeom KW . Radiomics in brain tumor: Image assessment, quantitative feature descriptors, and machine-learning approaches. Am J Neuroradiol. (2018) 39:208–16. doi: 10.3174/ajnr.A5391 28982791 PMC5812810

[13] Meng L Zhang R Fa L Zhang L Wang L Shao G . ATRX status in patients with gliomas: Radiomics analysis. Med U S. (2022) 101:E30189. doi: 10.1097/MD.0000000000030189 36123880 PMC9478307

[14] Nacul Mora NG Akkurt BH Kasap D Blömer D Heindel W Mannil M . Comparison of MRI sequences to predict ATRX status using radiomics-based machine learning. Diagnostics. (2023) 13:2216. doi: 10.3390/diagnostics13132216 37443610 PMC10341337

[15] Chung CYC Pigott LE . Predicting IDH and ATRX mutations in gliomas from radiomic features with machine learning: A systematic review and meta-analysis. Front Radiol. (2024) 4:1493824. doi: 10.3389/fradi.2024.1493824 39544481 PMC11560782

[16] Prysiazhniuk Y Server A Leske H Bech-Aase Ø Helseth E Eijgelaar RS . Diffuse glioma molecular profiling with arterial spin labeling and dynamic susceptibility contrast perfusion MRI: A comparative study. Neuro Oncol Adv. (2024) 6:vdae113. doi: 10.1093/noajnl/vdae113 39036439 PMC11259011

[17] Tomaszewski MR Gillies RJ . The biological meaning of radiomic features. Radiology. (2021) 298:505–16. doi: 10.1148/radiol.2021202553 33399513 PMC7924519

[18] UCSD-PTGBM . The Cancer Imaging Archive (Tcia). Available online at: https://www.cancerimagingarchive.net/collection/ucsd-ptgbm/ (Accessed September 14, 2026).

[19] Lundberg SM Lee SI . A unified approach to interpreting model predictions. Adv Neural Inf Process Syst. (2017) 30:4765–74.

[20] Zikou AK Romeo E Alexiou GA Lampros M Voulgaris S Astrakas L . The role of advanced MR imaging in gliomas. Appl Sci. (2026) 16:1027. doi: 10.3390/app16021027 30654563

[21] Van Cauter S Veraart J Sijbers J Peeters RR Himmelreich U De Keyzer F . Gliomas: Diffusion kurtosis MR imaging in grading. Radiology. (2012) 263:492–501. doi: 10.1148/radiol.12110927 22403168

[22] Kunimatsu A Yasaka K Akai H Sugawara H Kunimatsu N Abe O . Texture analysis in brain tumor MR imaging. Magn Reson Med Sci. (2021) 21:95–109. doi: 10.2463/mrms.rev.2020-0159 33692222 PMC9199980

[23] Vickers AJ Holland F . Decision curve analysis to evaluate the clinical benefit of prediction models. Spine J. (2021) 21:1643–8. doi: 10.1016/j.spinee.2021.02.024 33676020 PMC8413398

[24] Chawla N Bowyer K Hall L Kegelmeyer W . SMOTE: Synthetic minority over-sampling technique. J Artif Intell Res. (2002) 16:321–57. doi: 10.1613/jair.953

